# Supplementary material for: SEC-MX: an approach to systematically study the interplay between protein assembly states and phosphorylation
Source: Nat Commun. 2025 Jan 30;16:1176. doi: 10.1038/s41467-025-56303-0 (PMC11782603; doi:10.1038/s41467-025-56303-0)
Supplement: Supplementary file 1 — Supplementary Information [file 41467_2025_56303_MOESM1_ESM.pdf]

Supplementary Figures

Supplementary Figure 1 (Legend on next page):

A

HEK Mix Scheme

channel \ mix	mix1	mix2	mix3	mix4	mix5	mix6	mix7	mix8
126C	1	10	19	28	37	46	55	64
127N	64	1	10	19	28	37	46	55
127C	2	11	20	29	38	47	56	65
128N	65	2	11	20	29	38	47	56
128C	3	12	21	30	39	48	57	66
129N	66	3	12	21	30	39	48	57
129C	4	13	22	31	40	49	58	67
130N	67	4	13	22	31	40	49	58
130C	5	14	23	32	41	50	59	68
131N	68	5	14	23	32	41	50	59
131C	6	15	24	33	42	51	60	69
132N	69	6	15	24	33	42	51	60
132C	7	16	25	34	43	52	61	70
133N	70	7	16	25	34	43	52	61
133C	8	17	26	35	44	53	62	71
134N	71	8	17	26	35	44	53	62
134C	9	18	27	36	45	54	63	72
135	72	9	18	27	36	45	54	63

B

HEK-HCT Mix Scheme

channel \ mix	Cell-type	mix1	mix2	mix3	mix4	mix5	mix6	mix7	mix8	mix9	mix10	mix11	mix12
126	HEK	1	10	10	19	19	28	28	37	37	46	46	1
127N	HCT	1	10	10	19	19	28	28	37	37	46	46	1
127C	HEK	2	11	11	20	20	29	29	38	38	47	47	2
128N	HCT	2	11	11	20	20	29	29	38	38	47	47	2
128C	HEK	3	12	12	21	21	30	30	39	39	48	48	3
129N	HCT	3	12	12	21	21	30	30	39	39	48	48	3
129C	HEK	4	13	13	22	22	31	31	40	40	49	49	4
130N	HCT	4	13	13	22	22	31	31	40	40	49	49	4
130C	HEK	5	5	14	14	23	23	32	32	41	41	50	50
131N	HCT	5	5	14	14	23	23	32	32	41	41	50	50
131C	HEK	6	6	15	15	24	24	33	33	42	42	51	51
132N	HCT	6	6	15	15	24	24	33	33	42	42	51	51
132C	HEK	7	7	16	16	25	25	34	34	43	43	52	52
133N	HCT	7	7	16	16	25	25	34	34	43	43	52	52
133C	HEK	8	8	17	17	26	26	35	35	44	44	53	53
134N	HCT	8	8	17	17	26	26	35	35	44	44	53	53
134C	HEK	9	9	18	18	27	27	36	36	45	45	54	54
135	HCT	9	9	18	18	27	27	36	36	45	45	54	54

C

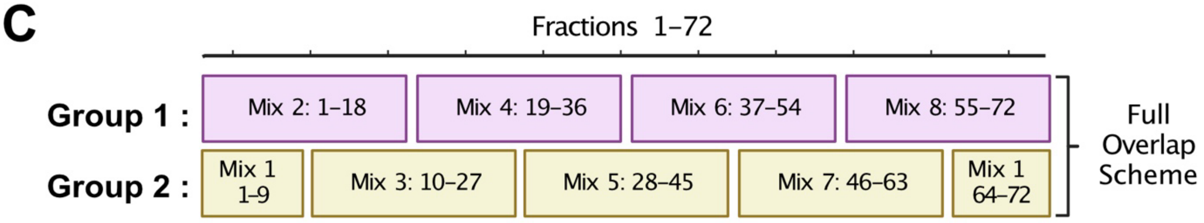

D

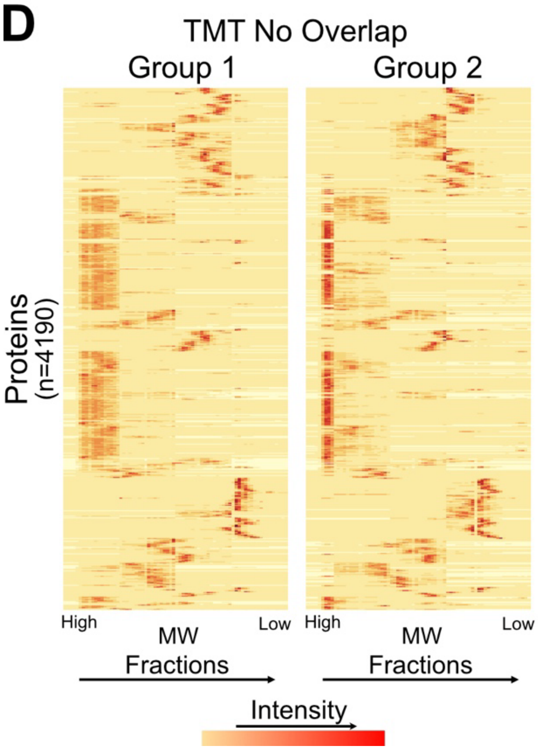

E

Condition	Set	Peptides	Proteins
MX	Full Overlap	69,036	6,152
	Group 1	56,095	5,668
	Group 2	57,088	5,737
DIA		90,254	6,748

F

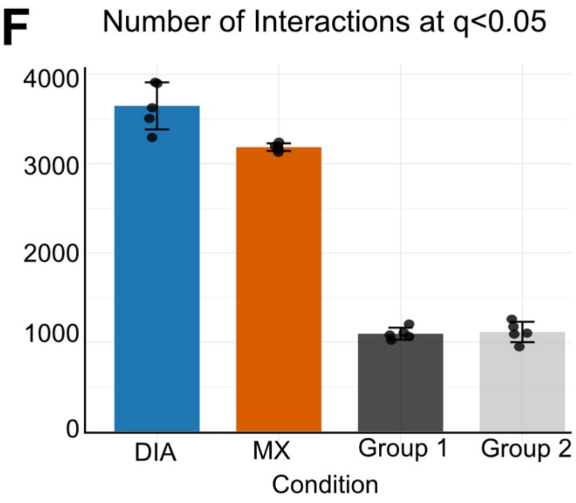

Supplementary Figure 1: Multiplexing schemes in developing SEC-MX (Previous page)

- **(A)** Label used (TMTpro18) per fraction and mix-pooling scheme in the dataset comparing SEC-MX to SEC-DIA.
- **(B)** Label used (TMTpro18) per fraction and mix-pooling scheme in the datasets used for gSEC and phSEC from HEK293 and HCT116 cells. gSEC replicate 1 was acquired using the no-overlap scheme (mixes 1,3,5,7,9,11), and gSEC replicate 2 was acquired using the full-overlap. Both replicates of phSEC were acquired with the no-overlap scheme using mixes 1,3,5,7,9,11.
- **(C)** A representation of the 72 collected fractions for the initial HEK293 TMT multiplexing. Group 1 consists of evenly numbered TMT mixes of all 72 fractions. Group 2 consists of the odd-numbered TMT mixes of all 72 fractions. The full-overlap scheme utilizes both TMT mixing groups – with all fractions measured twice, and mixes from both groups in an adjacent staggered fashion to allow for inter-mix normalization. After acquisition and signal processing fractions that were "empty" based on signal intensity were filtered out, resulting in 57 protein-containing fractions. "Created in BioRender. Jovanovic, M. (2024) https://urldefense.proofpoint.com/v2/url?u=https-3A__BioRender.com_s07v514&d=DwIFaQ&c=009klHSCxuh5Al1vNQzSO0KGjl4nbi2Q0M1QLJX9BeE&r=hhJH82V8klCl0kTThmjR5BtgUO1own1H2FZ90ykO1qs&m=eKSXGHrJ7-JNOXnNVO2A3ym1on69Vz0-JldKyzvICrRNKBxPkzbj2179L-D7Gkj3&s=D9pYHmOjP1iggQ8eu_yD-LBiQT0wUX0BTInRiHEGXnA&e= ".
- **(D)** Heatmap representation of the elution of proteins with a pseudo "no-overlap" scheme (n =4190): each group of mixes was searched on its own. Proteins identified in both groups were considered in this analysis in order to compare the elution patterns between the groups. Replicate 1 is shown. The rows in the two heatmaps are ordered the same. The intensities are max-normalized to 1 for each protein.
- **(E)** Number of peptide- and protein-identifications in both DIA and MX, with MX split between Group 1, Group 2, and Full-Overlap.
- **(F)** Bar graph representation of the number of interactions identified with SECAT at a q-value less than 0.05 for DIA, full overlap (MX), Group 1 (no-overlap MX), and Group 2 (no-overlap MX). The error bar represents the mean and standard deviation across replicates, and overlaid data points represent values measured in individual SECAT runs (n = 5).

Supplementary Figure 2 (Legend on next page):

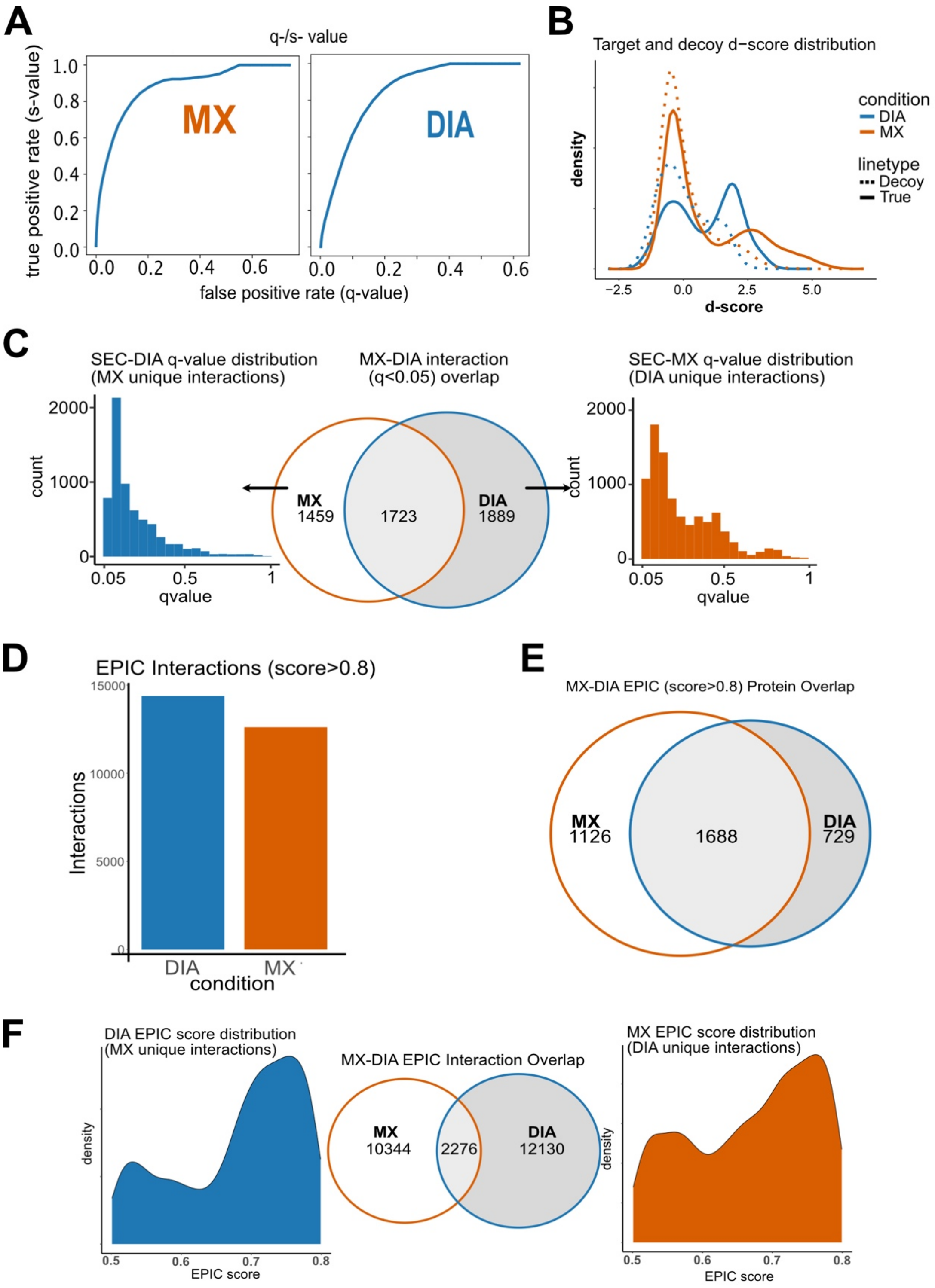

Supplementary Figure 2: Comparing SEC-DIA and SEC-MX by Interaction Analyses (Previous page)

- **(A)** Receiver-operating characteristic curve (ROC) of SEC-MX (left) and DIA (right) data by SECAT. The y-axis represents the true positive detection rate of (s-value). The x-axis represents the false positive detection rate (q-value).
- **(B)** SECAT results of detection of interactions between protein-protein pairs for either CORUM targets (solid lines) or decoys (dotted lines) by discriminant score (SECAT) for both SEC-DIA (Blue) and SEC-MX (Orange) data.
- **(C)** Overlap of SECAT identified interactions at a q-value less than 0.05. For interactions identified uniquely in one dataset but not the otehr, further q-value distribution plots for the opposite experiment are shown.
- **(D)** Barplot of the number of interactions identified using EPIC (n = 1) with a score cutoff of greater than 0.8.
- **(E)** Protein/node overlap between DIA and MX of the proteins identified in the EPIC interaction network.
- **(F)** Same as (C), for EPIC interactions passing a cutoff of 0.8 interaction score. The overlap was found to be statistically significant using a hypergeometric survival function (one-sided, log10(p-value) = -2,022)

Supplementary Figure 3 (Legend on next page):

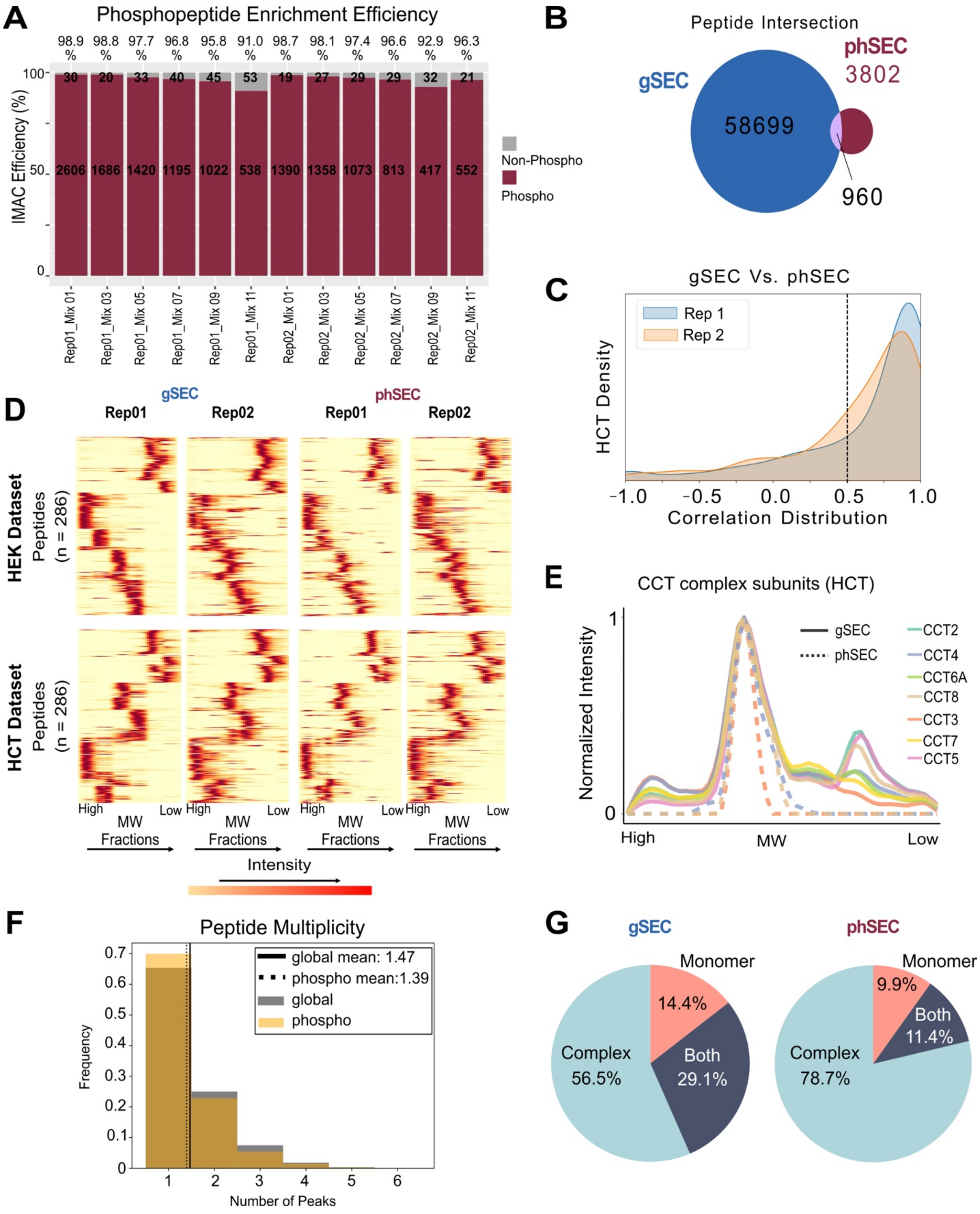

Supplementary Figure 3: Generating and comparing gSEC and phSEC datasets (Previous page)

(A) Phosphopeptide enrichment efficiency by Immobilized metal affinity chromatography (IMAC) for each TMT-mix used in phSEC. The numbers inside the bars represent the number of peptides identified in the mix with (Maroon) or without (gray) phospho(STY) modification. The numbers on top represent the calculated efficiency (in %).

(B) Venn diagram showing the overlap of peptides identified in both the gSEC and phSEC datasets (based on stripped sequences identified in the union of HEK293 and HCT116 cells).

(C) Distribution of Pearson correlation coefficients between gSEC and phSEC elution profiles for each overlapping peptide in HCT116, per replicate. In replicate 1, 471 out of 647 proteins have correlation coefficients greater than 0.5. In replicate 2, 362 out of 524 proteins have correlation coefficients greater than 0.5**.** Similar data from HEK293 cells is presented in main Figure 2C.

(D) Heatmaps of gSEC and phSEC by replicate for HEK293 data (top), HCT116 data (down). Signal is normalized to 1 by peptide maximum intensity and organized by the same dendrogram. The peptide number (n = 286) represents the number of rows and is based on the intersection of peptide identifications across replicates, conditions (HEK293/HCT116), and PTM (gSEC/phSEC).

(E) Elution profile plots for the CCT complex subunits in HCT116 (average intensity of replicates). Solid lines are from gSEC. The dashed lines are from phSEC. Subunits are color-coded as indicated in the key to the right of the trace-plots.

(F) Stacked bar plot histogram of the number of peaks identified per peptide in gSEC (gray) and phSEC (yellow).The solid and dotted line represents the mean number of peaks per peptide in gSEC and phSEC, respectively.

(G) Same as main figure 3C-D, only for the subset of proteins with identified phosphopeptides (n=1596 proteins). Pie charts show the percentage of proteins eluting exclusively as monomers, exclusively as complexed, or both. Peaks were identified based on peptide data and counted per protein.

Supplementary Figure 4 (Legend on next page):

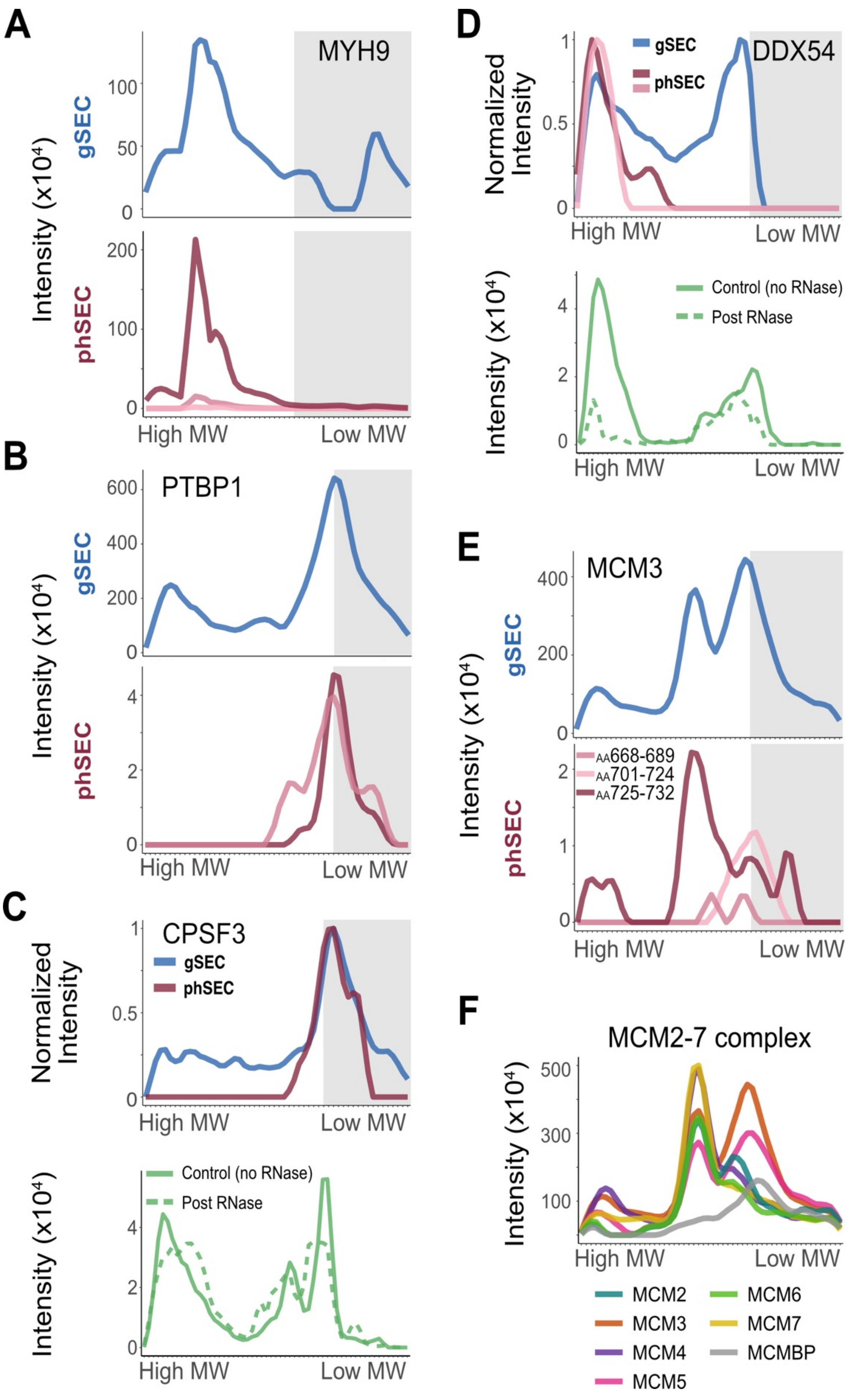

<u>Supplementary Data Figure 4: Additional Examples Supporting Main Figure 4, Mapping PTM onto assembly-states</u> <u>(Previous page)</u>

(A) Same as main figure 4C – but from HCT116 cells: SEC-MX elution traces in gSEC (top) and phSEC (bottom) for MYH9 in HCT116 cells (averaged across replicates). Gray boxes indicate the range of fractions covering the monomeric form of the protein. The three pink shaded traces in phSEC represent 3 different phosphopeptides all spanning the phosphorylation site on Serine-1943.

(B) Same as main figure 4D – but from HCT116 cells: SEC-MX elution traces in gSEC (top) and phSEC (bottom) for PTBP1 in HCT116 cells (averaged across replicates). Gray boxes indicate the range of fractions covering the monomeric form of the protein. The two pink shaded traces in phSEC represent 2 different phosphopeptides spanning the phosphorylation site on Serine-140 (localization probability = 0.5) or Serine-141 (localization probability = 0.5).

(C) Top: SEC-MX elution traces in gSEC (blue) and phSEC (maroon) for CPSF3 in HEK293 cells (averaged across replicates). Gray boxes indicate the range of fractions covering the monomeric form of the protein. Bottom: SEC-DIA signal for CPSF3 in HEK293 cells before (solid line) or after (dashed line) RNAse digestion. The maroon shaded trace in phSEC represent a phosphopeptides spanning the phosphorylation site on Threonine-681. **(D)** Same as C, for DDX54. The two pink shaded traces in phSEC represent 2 phosphopeptides spanning the phosphorylation site on Serine-782.

(E) Same as main figure 4F – but from HCT116 cells: SEC-MX elution traces in gSEC (top) and phSEC (bottom) for MCM3 in HCT116 cells (averaged across replicates). Gray boxes indicate the range of fractions covering the monomeric form of the protein.

(F) Replicate averaged elution profiles for MCM2-7 complex subunits (color-coded by subunit as indicated in the key at the bottom) in HCT116.

Supplementary Figure 5:

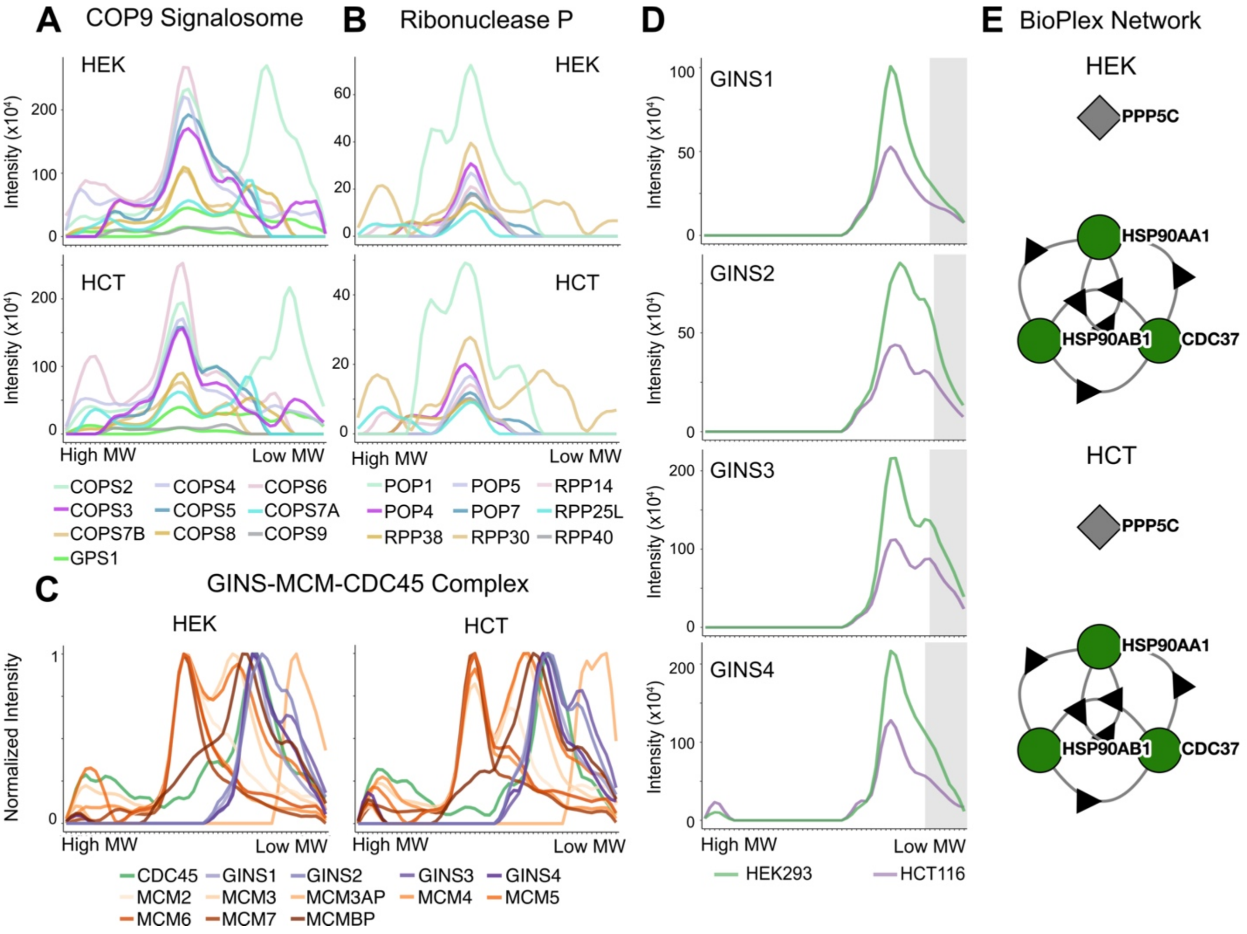

Supplementary Figure 5: SEC-MX enables differential comparison between biological samples

(A) Replicate averaged gSEC elution traces for COP9 signalosome complex in HEK293 (top) and HCT116 (bottom). Line colors indicate the different subunit of the complex as indicated in the key at the bottom.

(B) Replicate averaged gSEC elution traces for Ribonuclease P complex in HEK293 (top) and HCT116 (bottom). Line colors indicate the different subunit of the complex as indicated in the key at the bottom.

(C) Replicate averaged gSEC elution traces for the GINS-MCM-CDC45 complex in HEK293 (left) and HCT116 (right). Line colors indicate the different subunit of the complex as indicated in the key at the bottom.

(D) Replicate averaged gSEC elution traces for GINS complex subunits (GINS1-4, from top to bottom). HEK293 (green) and HCT116 (purple). Gray boxes indicate the range of fractions covering the monomeric form of the protein.

(E) BioPlex network of CDC37-HSP90 complex binary interactions as identified by AP-MS in the Bioplex network[43]. (As taken from the BioPlex online explorer tool: https://BioPlex.hms.harvard.edu/explorer/)

Supplementary Figure 6:

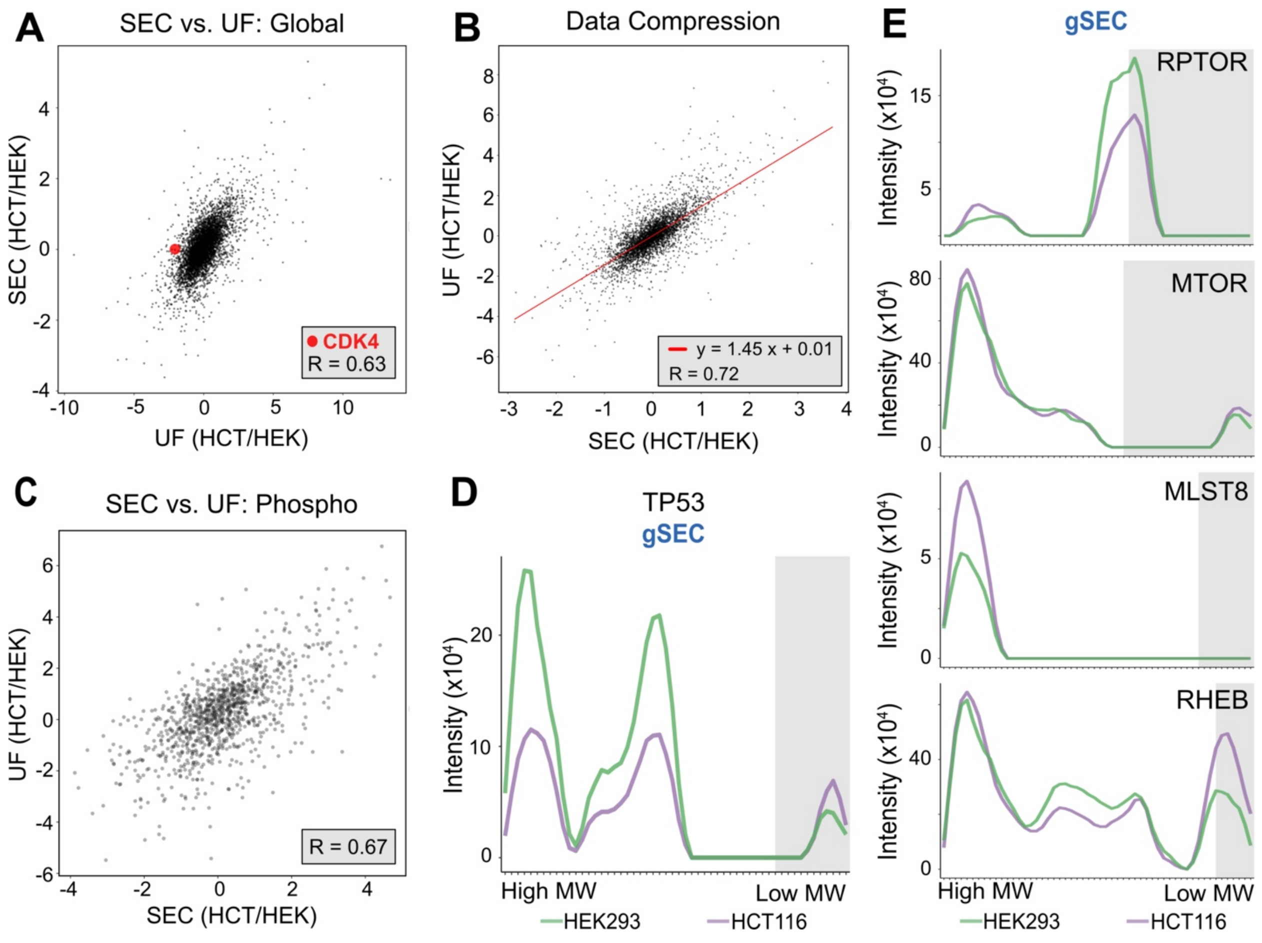

Supplementary Figure 6: Assembly-state resolution unveils differential analysis beyond abundance measurements

(A-B) Scatterplots comparing HCT116/HEK293 ratios in gUF (total intensity) to gSEC (either average of peak heights in A, or to sum of all fraction intensities in B). Each dot represents a protein. Note that these scatterplots can be compared to main figure 6A (single assembly-states in gSEC versus gUF). The change in R values (linear regression model) from 0.55 (individual peaks – main Figure 6A), to 0.63 (averaged peaks, panel A), to 0.72 (summed intensities, panel B), reveals a wider distribution of fold changes when comparing to distinct assembly-states, indicating there is additional information in the distinct assembly-states. The comparison in panel B was used to estimate TMT ratio compression, based on the assumption that the SEC aggregated signal can be compared to UF (unfractionated) signal. Ratio compression is calculated as 1/1.45.

(C) Scatterplot comparing HCT116/HEK293 ratios in phSEC (peak heights) versus phUF (total intensity). Each dot represents a protein assembly-state. R = 0.67 using a linear regression model.

(D) Replicate averaged gSEC elution traces for TP53. HEK293 (green) and HCT116 (purple). Gray boxes indicate the range of fractions covering the monomeric form of the protein.

(E) Replicate averaged gSEC elution traces for core members of the mTOR complex 1 (From top to bottom: RPTOR, MTOR, MLST8, RHEB). HEK293 (green) and HCT116 (purple). Gray boxes indicate the range of fractions covering the monomeric form of each protein.

Supplementary Figure 7:

A **Reactome Enrichment**

	Phospho ↑ Global –	Phospho ↑ Global ↑	Phospho – Global ↑
HCT	**B** RHO GTPases activate IQGAPs<br>mTORC1-mediated signaling<br>Signaling by ALK in cancer	**C** Viral mRNA Translation<br>Selenocysteine synthesis<br>Eukaryotic Translation Elongation	**F** Axon guidance<br>Nervous system development<br>Developmental Biology
HEK	**H** Diseases of programmed cell death<br>Cell Cycle<br>Transcriptional Regulation by TP53	**G** Regulation of MECP2 expression and activity<br>Signaling by NOTCH<br>Regulation of PTEN gene transcription	**D** Signaling by NOTCH3<br>mRNA splicing<br>SUMOylation

B **Kinase Target Enrichment**

	Phospho ↑ Global –	Phospho ↑ Global ↑	Phospho – Global ↑
HCT	**B** • RPS6KB1<br>• MTOR<br>• MAPK3	**C** • PRKACA<br>• PRKCA<br>• PRKCI	**F** • PLK3<br>• MAPK14<br>• AURKB
HEK	**H** • ATR<br>• ATM<br>• CHEK2	**G** • PRKCE<br>• MAPK10<br>• PRKCD	**D** • PRKCE<br>• ATR<br>• PRKCD

Supplementary Figure 7: Using SEC-MX to explore functional pathways

(A) Table of Reactome Pathway Enrichment for 6 out of the 9 response groups described in main Figure 7A-B, as determined by WebGestalt. The top 3 pathways (based on enrichment ratios) are shown per group (p-value < 0.05).

(B) Kinase prediction by kinase target enrichment analysis[51] results for 6 out of the 9 response groups described in 7A-B. The top 3 kinases are shown per group (p-value < 0.05).

Supplementary Figure 8:

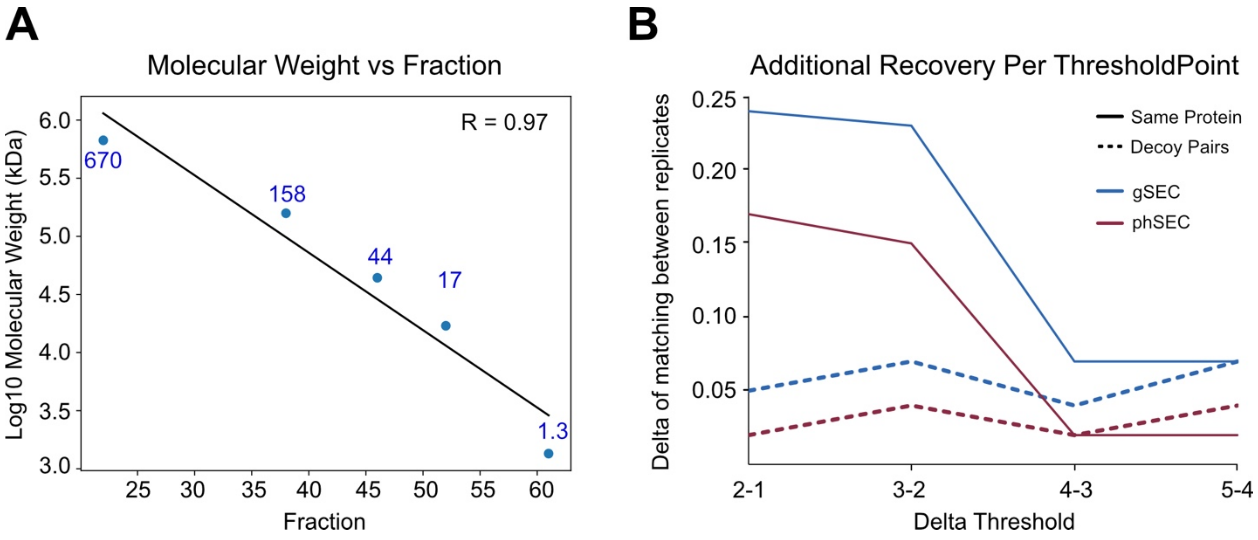

Supplementary Figure 8: supporting information for the data analysis section (under Methods)

(A) Plot of molecular weight (MW) estimation (y-axis) per fraction (x-axis) based on a MW standard (Biorad, cat# 1511901) that was run as a quality-control together with the HEK293-HCT116 SEC runs. The y-axis is the log10 of the MW (kDa), due to the log-linear relationship between fractions and MW. The predicted MW of each standard component is marked in blue. This estimation is limited to the range of fractions where the standard molecules eluted and therefore starts in fraction 21 (estimated 670 kDa) and extends beyond the range of protein-containing fractions (which was fraction 54). As shown, the correlation was calculated using a linear regression model resulting in R = 0.97.

(B) Plot depicting the optimization process for determining the threshold distance (in fractions) for peak alignment (where aligned peaks indicate matching between proteins in gSEC and peptides in phSEC). The threshold is the maximum distance in fractions between two peaks whereby an alignment can be called. The increase in threshold shows the value provided by increase the threshold from 1 to 2 (2-1), 2 to 3 (3-2), and so on (x-axis). The y-axis indicates the average increase in peak-alignment (recovery) between the elution-peaks in two distinct replicates: either between the two replicates of the same protein/phosphopeptide (Same Protein, solid line) or a random pair (Decoy Pair, dashed line). The y-axis value takes the average of these values for all the profiles it calculated on, and then subtracts the average between the two threshold cutoffs – thereby showing the increase in peak alignment provided by increasing the threshold. This was done for both global proteins (gSEC, blue) and phosphopeptides (phSEC, maroon) data. A threshold of 3 was chosen since increasing the threshold to 4 provided the same increase in alignment as the decoy pairs.

Supplementary Tables

Supplementary Table 1:
Overview of assembly-state relevant identifications per dataset and replicate

Groups			Numbers				
Replicate	Condition	Dataset	Proteins	Peptides	Pep Peaks	Prot Peaks	Assembly States
01	HCT	gSEC	4,578	41,429	57,234	7,747	8,519
		phSEC	2,027	4,237	6,314		
	HEK	gSEC	4,583	41,466	52,236	7,083	
		phSEC	1,993	4,159	5,473		
02	HCT	gSEC	5,154	46,763	69,702	9,508	11,592
		phSEC	1,477	2,745	4,101		
	HEK	gSEC	5,151	46,743	71,240	10,005	
		phSEC	1,483	2,761	3,837		
