# Supplementary material for: SEC-MX: an approach to systematically study the interplay between protein assembly states and phosphorylation
Source: Nat Commun. 2025 Jan 30;16:1176. doi: 10.1038/s41467-025-56303-0 (PMC11782603; doi:10.1038/s41467-025-56303-0)
Supplement: Supplementary file 2 — Description of Additional Supplementary Files [file 41467_2025_56303_MOESM2_ESM.pdf]

Description of Additional Supplementary Files

File Name: Supplementary Data 1

Description: Processed signal per fraction in gSEC (global) ofHEK293 and HCT116 cells. Data is normalized, smoothed, and replicate-averaged. Missing values converted to 0. Data is given at the protein level, columns labeled 1-54 correspond to the SEC fractions

File Name: Supplementary Data 2

Description: Processed signal per fraction in phSEC (enriched phosphopeptides) of HEK293 and HCT116 cells. Data is normalized, smoothed, and replicate-averaged. Missing values converted to 0. Data is given at the peptide level (stripped sequence), columns labeled 1-54 correspond to the SEC fractions.

File Name: Supplementary Data 3

Description: Detailed lists of the proteins with peaks unique in each cell-line's dataset or mutual to both. In gSEC and phSEC separately. Data given as GeneNames.

File Name: Supplementary Data 4

Description: Intensity values from unfractionated samples (UF) - not normalized, not imputed, averaged across replicates. Data for both global (gUF, ptm = global) and phosphopeptide enriched (phUF, ptm = phospho).

File Name: Supplementary Data 5

Description: Detailed lists of the proteins with peaks falling in each one of the 9 response groups defined by the scatterplot in Figure 7A. Data given as GeneNames.
