# Supplementary material for: SEC-MX: an approach to systematically study the interplay between protein assembly states and phosphorylation
Source: Nat Commun. 2025 Jan 30;16:1176. doi: 10.1038/s41467-025-56303-0 (PMC11782603; doi:10.1038/s41467-025-56303-0)
Supplement: Supplementary file 8 — Reporting Summary [file 41467_2025_56303_MOESM8_ESM.pdf]

nature portfolio

Corresponding author(s): Marko Jovanovic & Ella Doron-Mandel

Last updated by author(s): Dec 4, 2024

Reporting Summary

Nature Portfolio wishes to improve the reproducibility of the work that we publish. This form provides structure for consistency and transparency in reporting. For further information on Nature Portfolio policies, see our Editorial Policies and the Editorial Policy Checklist.

Statistics

For all statistical analyses, confirm that the following items are present in the figure legend, table legend, main text, or Methods section.

n/a	Confirmed	
☐	☒	The exact sample size (*n*) for each experimental group/condition, given as a discrete number and unit of measurement
☐	☒	A statement on whether measurements were taken from distinct samples or whether the same sample was measured repeatedly
☐	☒	The statistical test(s) used AND whether they are one- or two-sided<br>*Only common tests should be described solely by name; describe more complex techniques in the Methods section.*
☒	☐	A description of all covariates tested
☐	☒	A description of any assumptions or corrections, such as tests of normality and adjustment for multiple comparisons
☐	☒	A full description of the statistical parameters including central tendency (e.g. means) or other basic estimates (e.g. regression coefficient) AND variation (e.g. standard deviation) or associated estimates of uncertainty (e.g. confidence intervals)
☐	☒	For null hypothesis testing, the test statistic (e.g. *F*, *t*, *r*) with confidence intervals, effect sizes, degrees of freedom and *P* value noted<br>*Give P values as exact values whenever suitable.*
☒	☐	For Bayesian analysis, information on the choice of priors and Markov chain Monte Carlo settings
☒	☐	For hierarchical and complex designs, identification of the appropriate level for tests and full reporting of outcomes
☐	☒	Estimates of effect sizes (e.g. Cohen's *d*, Pearson's *r*), indicating how they were calculated

Our web collection on statistics for biologists contains articles on many of the points above.

Software and code

Policy information about availability of computer code

Data collection	Liquid chromatography tandem mass-spectrometry acquisition was conducted on a Q-exactive HF (Thermo Fisher Scientific) with vendor provided software (Xcalibur 4.5).

Data analysis

Only open source versions were used in the analysis:

Mass Spectrometry Analysis:
All DDA/TMT raw files were analyzed by SpectroMine (Version 4.4.231122.52329, Lapis lazuli). All DIA raw files were analyzed by Spectronaut (Version 17.1.221229.55965, Quasar).

Preprocessing and Peak Alignment:
Package versions and code available from Github (https://doi.org/10.5281/zenodo.14219108) (https://github.com/mjlab-Columbia/SPADE-PTM)

Protein Interaction Analyses:
SECAT (version 1.1.5) from Github (https://github.com/grosenberger/secat)
EPIC from Github (https://github.com/BaderLab/EPIC)

Enrichment Analysis:
WebGestalt from (https://www.webgestalt.org/)

For manuscripts utilizing custom algorithms or software that are central to the research but not yet described in published literature, software must be made available to editors and reviewers. We strongly encourage code deposition in a community repository (e.g. GitHub). See the Nature Portfolio guidelines for submitting code & software for further information.

Data

Policy information about availability of data

All manuscripts must include a data availability statement. This statement should provide the following information, where applicable:

- Accession codes, unique identifiers, or web links for publicly available datasets
- A description of any restrictions on data availability
- For clinical datasets or third party data, please ensure that the statement adheres to our policy

Original data:
Mass spectrometry raw files and preprocessed data have been deposited to the ProteomeXchange Consurtium via the MassIVE partner repository with identifier MSV000096001 and MSV000093915.

Reference datasets and databases:
UniprotKB/Swiss-Prot Human FASTA downloaded from https://www.uniprot.org/ (organism id 9606).
Phosphosite data and kinase prediction were retrieved from PhosphoSitePlus at https://www.phosphosite.org/homeAction.action.
Protein structure data was obtained from the protein data bank (https://www.rcsb.org/)
Gene ontology, reactome pathways and kinase target enrichment were conducted via webGestalt at https://www.webgestalt.org/
Protein interaction database: String PPI DB (version 11.5, https://string-db.org/), corum (https://mips.helmholtz-muenchen.de/corum/)

Research involving human participants, their data, or biological material

Policy information about studies with human participants or human data. See also policy information about sex, gender (identity/presentation), and sexual orientation and race, ethnicity and racism.

Reporting on sex and gender	N/A
Reporting on race, ethnicity, or other socially relevant groupings	N/A
Population characteristics	N/A
Recruitment	N/A
Ethics oversight	N/A

Note that full information on the approval of the study protocol must also be provided in the manuscript.

Field-specific reporting

Please select the one below that is the best fit for your research. If you are not sure, read the appropriate sections before making your selection.

☒ Life sciences ☐ Behavioural & social sciences ☐ Ecological, evolutionary & environmental sciences

For a reference copy of the document with all sections, see nature.com/documents/nr-reporting-summary-flat.pdf

Life sciences study design

All studies must disclose on these points even when the disclosure is negative.

Sample size	Due to the considerable resources needed and efforts required for data collection, we kept the number of biological replicates to the minimum required in order to verify reproducibility of the results.
Data exclusions	For HEK293 versus HCT116 SEC-MX experiments we initially acquired a third biological replicate. However, the input amounts required for generating the phSEC dataset required consolidating 2 replicates in order to achieve the satisfactory level of data quality and therefore, gSEC replicate 3 was omitted from the final analysis in order to match to phSEC replicates.
Replication	Due to the large scale of the experiments, no technical replicates were acquired.
Randomization	No randomization was used due to the consecutive nature of SEC fractions.
Blinding	Blinding was not relevant in this study as it was conducted as an unbiased discovery effort.

Reporting for specific materials, systems and methods

We require information from authors about some types of materials, experimental systems and methods used in many studies. Here, indicate whether each material, system or method listed is relevant to your study. If you are not sure if a list item applies to your research, read the appropriate section before selecting a response.

Materials & experimental systems

n/a	Involved in the study
☒	☐ Antibodies
☐	☒ Eukaryotic cell lines
☒	☐ Palaeontology and archaeology
☒	☐ Animals and other organisms
☒	☐ Clinical data
☒	☐ Dual use research of concern
☒	☐ Plants

Methods

n/a	Involved in the study
☒	☐ ChIP-seq
☒	☐ Flow cytometry
☒	☐ MRI-based neuroimaging

Eukaryotic cell lines

Policy information about cell lines and Sex and Gender in Research

Cell line source(s)	HEK293XT cells (Takara Bio Lenti-X 293T, #632180) were provided by the Yeo lab at UC San Diego (SEC-DIA versus SEC-MX experiments), or purchased from ATCC (ATCC, CRL-3216, in HEK293 versus HCT116 experiments), HCT116 cells were provided by the Prives lab at Columbia University. HEK293 cells are derived from a female human fetus, HCT116 cells are derived from adult male.
Authentication	No authentication was conducted.
Mycoplasma contamination	Cells were monitored routinely for mycoplasma contamination every 5-10 passages and were found clean.
Commonly misidentified lines (See ICLAC register)	N/A

Plants

Seed stocks	N/A
Novel plant genotypes	N/A
Authentication	N/A
